# Skilled nursing facility wastewater surveillance: a SARS-CoV-2 and antimicrobial resistance detection pilot study

**DOI:** 10.2166/wh.2025.374

**Published:** 2025-05-09

**Authors:** Ariel Jose Santiago, Maria Burgos Garay, Mariya Campbell, Yimu Cahela, Rodney Donlan, Paige Gable, Christine Ganim Kyros, Lauren Franco, Leila Kartforosh, Susanna Lenz, Amanda K Lyons, Jamari Moore, Judith Noble-Wang, Carrie Sanders, Bethelhem Abera, Colin H. Adler, Sophie Jones, Magdalena Medrzycki, Maroya S. Walters, Peter Cook, Yan Li, Ying Tao, Jing Zhang, Lakshmi Malapati, Adam Retchless, Suxiang Tong, Angela D. Coulliette-Salmond

**Affiliations:** a Division of Healthcare Quality Promotion, National Center for Emerging and Zoonotic Infectious Diseases, Centers for Disease Control and Prevention, 1600 Clifton Rd., Atlanta, GA 30329, USA; b Oak Ridge Institute for Science and Education (ORISE), 1299 Bethel Valley Rd., Oak Ridge, TN 37830, USA; c Goldbelt C6 LLC, 860 Greenbrier Cir., #405, Chesapeake, VA 23320, USA; d Tanaq Support Services LLC, 3201 C St., Suite 602, Anchorage, AK 99503, USA; e Epidemic Intelligence Service, Centers for Disease Control and Prevention, 1600 Clifton Rd., Atlanta, GA 30329, USA; f United States Public Health Service, 1101 Wootton Pkwy., Plaza Level, Rockville, MD 20853, USA; g Coronavirus and Other Respiratory Viruses Division, National Center for Immunization and Respiratory Diseases, Centers for Disease Control and Prevention, 1600 Clifton Rd., Atlanta, GA 30329, USA

**Keywords:** antimicrobial resistance, SARS-CoV-2, skilled nursing facility, wastewater surveillance

## Abstract

The purpose of this study was to determine the feasibility of facility-level wastewater surveillance in the detection of severe acute respiratory syndrome coronavirus 2 (SARS-CoV-2) in skilled nursing facility (SNF) wastewater using three concentration methods, as well as a proof-of-concept for antimicrobial resistance (AR) genes/organisms detection. Wastewater effluent samples were collected from an SNF over an 8-week period. Wastewater was concentrated using electronegative membrane filtration (enMF), polyethylene glycol precipitation, and Nanotrap^®^ magnetic virus particles (NP). Quantification of the genome copy concentration from SARS-CoV-2 and bovine respiratory syncytial virus (BRSV), a SARS-CoV-2 surrogate spiked into all samples, was performed with droplet digital polymerase chain reaction (ddPCR). Wastewater sample aliquots were also enriched in microbiological culture media and screened for organisms with AR phenotypes on selective and differential agars. Multiplex real-time PCR was used to detect a broad array of carbapenem resistance genes. SARS-CoV-2 was detected and quantified from a single enMF-concentrated wastewater sample. The highest concentration of BRSV came from enMF-concentrated samples. *Klebsiella*, *Enterobacter*, *Citrobacter*, and *Escherichia coli* exhibiting AR phenotypes were successfully detected using culture-dependent approaches. Culture-independent, multiplex PCR indicated that bla_*KPC*_ was the main carbapenemase gene detected in wastewater samples. Facility-level wastewater surveillance could be a useful strategy for SNFs.

## Graphical Abstract

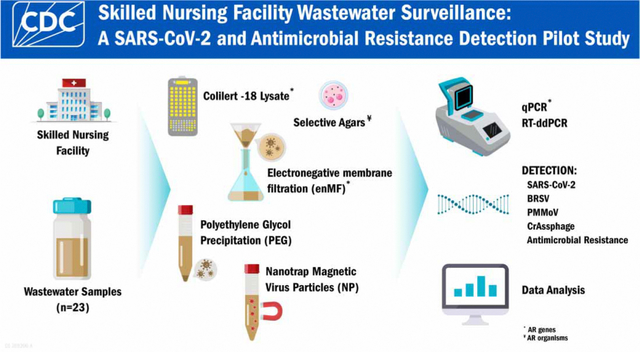

## INTRODUCTION

Wastewater-based epidemiology (WBE) has been a valuable public health surveillance tool used for monitoring the consumption of pharmaceuticals, illicit drugs, poliovirus, as well as exposure to chemical and other biological agents of public health concern (Orive et al. 2020; Huizer et al. 2021). During the global outbreak of the beta-coronavirus, severe acute respiratory syndrome coronavirus 2 (SARS-CoV-2), the use of WBE and associated wastewater surveillance methods proved to be a complementary monitoring tool in estimating real-time infection and coronavirus disease 2019 (COVID-19) burden in the community (Medema et al. 2020; Wu et al. 2020). SARS-CoV-2 genetic material and antimicrobial resistance (AR) genes/organisms can be shed through stool and remain detectable in wastewater both at the community-level (i.e., wastewater treatment plants) and at the facility level (e.g., primary schools, universities, and hospitals) (Wang et al. 2005; Acosta et al. 2021; Colosi et al. 2021; Harris-Lovett et al. 2021; Scott et al. 2021; Fielding-Miller et al. 2023). Wastewater surveillance in congregate settings, primarily university dormitories, has demonstrated its utility for the detection of SARS-CoV-2 (Gibas et al. 2021; Corchis-Scott et al. 2023). In the case of skilled nursing facilities (SNFs), residents were some of the most impacted individuals during the spread of SARS-CoV-2 (Abrams et al. 2020; Lau-Ng et al. 2020). SNF residents are a high-risk, high-acuity population typically having underlying, chronic medical conditions (D’Adamo et al. 2020), which likely contributed to the high mortality rates recorded during the early stages of the pandemic. As a result, several monitoring strategies, including testing of residents and healthcare workers coupled with facility-level wastewater surveillance, have been evaluated and used to inform guidelines for protecting these high-risk populations (Lee et al. 2021; Shrader et al. 2021; Tsoungui Obama et al. 2021). These enhanced surveillance strategies may also be extended to monitoring AR, an additional public health concern impacting SNFs. Determining success for detecting SARS-CoV-2 and antimicrobial-resistant threats at the SNF level and other long-term care facility equivalents has the potential for actionable results with public health impact.

Wastewater surveillance methods can provide trend data for specific targets in communities, university/college campuses, and prisons, representing hundreds to hundreds of thousands of individuals. Capturing the pathogen signal in a resident/patient population at an SNF also presents challenges due to the population being in the tens to hundreds and with only a limited portion of the population being mobile and capable of using the toilet and conducting hygienic practices (e.g., bathing or showering, sink use). Additionally, the erratic, low-flow effluent from the building contains debris and fresh residues from laundry, kitchen, etc. that can impact the detection of the target microorganism. While there are a few hospital wastewater surveillance studies published that demonstrate associations between SARS-CoV-2 detected in wastewater and clinical cases (Wang et al. 2005; Acosta et al. 2021; Colosi et al. 2021), there are only two peer-reviewed published studies currently available regarding SARS-CoV-2 detection in United States nursing homes (Gamage et al. 2024; Keck et al. 2024). In the work of Gamage et al. (2024), grab samples were collected and concentrated using centrifugation and ultrafiltration methods to demonstrate 60% sensitivity in detecting a SARS-CoV-2 infected resident. A study by Keck et al. study (2024) utilized 24 h composite samples and exclusion-based sample preparation to demonstrate a 48% sensitivity for identifying SARS-CoV-2 infections (Keck et al. 2024). An additional study conducted outside the United States applied an aluminum adsorption-precipitation concentration method (Davó et al. 2021). There is limited and inconsistent application of successful wastewater surveillance methods at the congregate-level to evaluate recovery for SARS-CoV-2 (CDC 2023a), and multiple studies in this setting are important to determine long-term feasibility.

In the few peer-reviewed papers regarding wastewater surveillance for AR genes, where five different isolation and detection approaches were taken, all the studies were conducted outside the United States (White et al. 2016; Buelow et al. 2018; Cahill et al. 2019; Buelow et al. 2020; Kagambèga et al. 2023). Implementing wastewater surveillance for AR organisms and genes in a SNF population or long-term care equivalent within the United States has yet to be thoroughly evaluated. A recent report by the National Academies of Sciences on wastewater-based disease surveillance highlighted the importance of facility-level testing as a more effective form of disease surveillance compared to community-wide surveillance, particularly when it comes to determining the spread of antibiotic resistance (National Academies of Sciences & Medicine 2024). Differing from SARS-CoV-2, where detection of the virus indicates an infection, detection of a bacterial pathogen and associated antimicrobial genes is complicated by colonized individuals contributing to this signal and even the natural environmental reservoir, not necessarily an active infection. If wastewater surveillance for AR is possible at SNFs in order to supplement infrequent point prevalence surveys, the gains in understanding would outweigh the challenges faced in attempting to reduce the estimated 35,000 deaths, 2.8 million infections, and $4.1–$5.1 billion spent in the United States per year with increased surveillance (CDC 2019). The AR focused targets for this project were the carbapenem genes *bla*_KPC_, *bla*_NDM_, *bla*_VIM_, and *bla*_OXA-like_, as these genes are responsible for a majority of resistant infections in facilities with high risk, high acuity populations and are recognized global AR threats.

This paper describes a pilot application of wastewater surveillance at an SNF in the Atlanta-metro area during the fall of 2021, with a primary focus on evaluating three methods for concentrating wastewater samples followed by DNA/RNA extraction and detection/quantification of genetic material from SARS-CoV-2, and endogenous and exogenous virus surrogates. Two of the three methods, electronegative membrane filtration (enMF) and polyethylene glycol precipitation (PEG), have traditionally been used for concentrating viruses from a variety of environmental matrices, including wastewater (La Rosa et al. 2020; Wu et al. 2020). The third, Nanotrap^®^ magnetic virus particles (NP), is a more recent technological development for capturing viral particles in wastewater and environmental matrices and was included to evaluate how it compares to the more traditional methods. Additionally, enriched wastewater samples were assessed for the presence of carbapenem resistance genes and AR Gram-negative Enterobacterales using selective agars and PCR assays typically used for clinical isolates. These findings will help to further inform potential approaches and considerations for wastewater surveillance, particularly at SNFs.

## MATERIALS AND METHODS

This study was approved by the CDC COVID-19 Response Laboratory Task Force and met the CDC safety policies and recommendations in the CDC/NIH *Biosafety in Microbiological and Biomedical Laboratories* (Meechan & Potts 2020). This activity was reviewed by CDC and was conducted consistent with applicable federal law and CDC policy.^§^

### Facility description and field sampling

Wastewater effluent samples were collected from a 200-bed SNF in the Atlanta-metro area (Georgia, USA) from September to November 2021. A survey at enrollment and eight weekly questionnaires were conducted to collect facility-level and epidemiological data (e.g., census, wastewater access), the number of residents with an active or within 30 days of a SARS-CoV-2 infection, and the number of confirmed SARS-CoV-2-infected residents who were able to use the toilet. Three of the weekly questionnaires were not able to be collected due to staffing turnover. At the beginning of enrollment in this study, the SNF had 168 residents and 181 healthcare personnel, with 88 and 74%, respectively, fully vaccinated against COVID-19 (fully vaccinated defined as receiving a 2-dose mRNA series or single dose Janssen vaccine ≥ 14 days prior) (CMMS 2021). The SNF had a designated 5-bed COVID-19 unit. Clinical services provided at this SNF included wound, ostomy, and indwelling medical device care. During this pilot, on two of the 8 weeks, resident toilet-use in the facility was measured at 21% (35 of 164) and 40% (66 of 164), potentially contributing to the wastewater effluent.

Twenty-four hour composite samples (*n* = 18) and grab samples (*n* = 5) were collected from a manhole outside of the SNF building using an Avalanche Portable Refrigerated Autosampler (Teledyne ISCO, Lincoln, NE, USA) (McHugh 2023). When possible, flow-based composite samples were collected using an Avalanche add-on ISCO 730 Bubbler Flow Module connected to a flow meter insert (Teledyne ISCO, Lincoln, NE, USA). Time-based composite samples were collected in lieu of flow-based, whenever heavy debris present in the wastewater effluent caused accuracy errors. Grab samples were collected when neither flow nor time-based samples were possible. Composite samples were collected four times per week as possible, based on laboratory workflow and personnel capacity.

### Wastewater controls

Four wastewater controls were used in this study. Bovine respiratory syncytial virus (BRSV) (Inforce3 Respiratory Vaccine, Zoetis Inc., Kalamazoo, MI USA) and human coronavirus OC43 (OC43) (ATCC, Manassas, VA USA) served as exogenous controls and pepper mild mottle virus (PMMoV) (CDC, Atlanta, GA USA) and the cross-assembly phage *Carjivirus communis* (Turner et al. 2023) (herein referred to as CrAssphage) (IDT, Coralville, IA USA) served as endogenous controls. BRSV was prepared according to manufacturer instructions and served as a whole process control and a virus surrogate for SARS-CoV-2. BRSV was spiked into wastewater samples prior to concentration methods. OC43 was prepared according to manufacturer instructions and served as an independent extraction control and was spiked into concentrated samples prior to RNA extraction. PMMoV and CrAssphage served as human fecal indicators.

A synthetic 2019_nCoV RNA construct (CDC, Atlanta, GA USA) was used as a positive control to ensure proper amplification and detection during droplet digital PCR (ddPCR). In addition, customized gene fragments were obtained for the endogenous controls PMMoV (CDC, Atlanta, GA USA) (Table S1A-Supplementary Information) and CrAssphage (IDT, Coralville, IA USA) (Table S1A-Supplementary Information), and prepared according to manufacturer instruction. All controls were included in each ddPCR assay and were run using two different dilutions (varied according to control) to check for the presence of PCR inhibitors (Loeb et al. 2020). All standard control nucleic acid concentrations were determined using ddPCR. To avoid degradation, all controls were prepared as single-use aliquots and stored at −80 °C for later use.

### Wastewater sample preparation: Pre-concentration

Composite wastewater samples were stored at 4 °C for 18–24 h with tracked, chain-of-custody once collected in the field. Wastewater (400 ml) was transferred to a sterile, 500 ml Nalgene bottle (containing a magnetic stir bar) and spiked with 400 μl of BRSV at a mean concentration (min-max) of 1.16 × 10^1^ gc/μl (0.31–47.20), as a whole process control. A negative control, herein referred to as blank, consisted of a 300 ml aliquot of autoclaved tap water (ATW), which was transferred to a sterile, 500 ml Nalgene bottle (containing a magnetic stir bar) and spiked with 300 μl of nuclease-free water. Wastewater and blank bottles were mixed at 120 rpm for 30 min (Barnstead/Thermolyne, Dubuque, IA USA). After mixing, the wastewater and blank samples were split to evaluate three separate concentration methods. Aliquots of 100 ml were transferred to 250 ml Nalgene bottles for PEG and enMF methods, while an aliquot of 40 ml was transferred to a 50 ml conical tube for the nanoparticle method (NP). The remaining wastewater was held at 4 °C for 24 h in the event replication was needed for any assay.

### Virus concentration methods

#### PEG precipitation

PEG precipitation was performed as described elsewhere with some modification (Ye et al. 2016; Ahmed et al. 2020a, 2020b; Graham et al. 2021). PEG 8000 (Fisher Scientific, Waltham, MA, USA) and sodium chloride (Fisher Scientific, Waltham, MA USA) were added to both the 100 ml blank (ATW + nuclease-free water) and wastewater samples at concentrations of 8% (w/v) and 0.2 M (final), respectively. Blank and wastewater sample bottles were incubated statically at 4 °C for 2 h then concentrated by centrifugation for 30 min at 20,000 ×*g* at 4 °C. The supernatant was discarded, and pellet re-suspended in 2.5 ml 1X phosphate buffered saline (PBS). Prior to viral RNA extraction, the wastewater sample was spiked with 200 μl of OC43 as an extraction control, while blank samples were spiked with 200 μl of nuclease-free water. Samples were briefly mixed by pipetting and 200 μl were transferred to 2 ml microcentrifuge tubes in preparation for RNA extraction.

#### Nanotrap^®^ magnetic virus particles (NPs)

Concentration by magnetic NPs was performed according to manufacturer suggestions with slight modification of sample volumes processed and RNA extraction kits used (Patnaik et al. 2020; Rasile 2021). Briefly, blank (ATW + nuclease-free water) and wastewater samples were transferred to separate 50 ml conical tubes in 40 ml aliquots and allowed to sit for 10 min at room temperature. Six hundred microliters (150 μl/10 ml sample) of Ceres Nanotrap^®^ Magnetic Virus Particles (currently Nanotrap^®^ Microbiome A Particles) (Ceres Nanosciences, Manassas, VA USA) were added to both blank and wastewater samples and mixed by gently inverting 2–3 times, followed by incubation for 20 min at room temperature. Following incubation, tubes were placed in a DynaMag^™^ 50 magnetic rack (Thermo Fisher Scientific, Waltham, MA, USA) for 20 min at room temperature to separate NPs from the samples. The supernatant was removed, and NPs were re-suspended in 200 μl of 1X PBS. Prior to viral RNA extraction, the wastewater sample was spiked with 200 μl of OC43 as a molecular extraction control, while blank samples were spiked with 200 μl of nuclease-free water. Samples were briefly mixed by pipetting and 200 μl were transferred to 2 ml microcentrifuge tubes in preparation for RNA extraction.

#### Electronegative membrane filtration

enMF was performed as described elsewhere, with the following modifications (Ahmed et al. 2020a). Magnetic filter funnels (Pall Corporation, Port Washington, NY, USA) were mounted onto a multi-position filter manifold (MilliporeSigma, Burlington, MA, USA) and loaded with 0.45 μm pore-size, 47-mm diameter enMFs (Fisher Scientific, Waltham, MA, USA). Filters were pre-moistened with 5 ml 1X PBS. Magnesium chloride (MgCl_2_-6H_2_O) (Fisher Scientific, Waltham, MA, USA) was added to 100 ml of both blank (ATW + nuclease-free water) and wastewater samples to a final concentration of 25 mM. Using a serological pipette, 100 ml of blank and wastewater samples were transferred and passed through the pre-moistened filter funnels. The enMFs were aseptically transferred to 2 ml PowerBead, bead beating tubes, containing 0.70 mm garnet beads (Qiagen, Germantown, MD, USA). Prior to viral RNA extraction, the wastewater sample was spiked with 200 μl of OC43 as a molecular extraction control, while blank samples were spiked with 200 μl of nuclease-free water in preparation for RNA extraction.

#### Viral nucleic acid extraction

Viral nucleic acid was extracted using the AllPrep^®^ PowerViral^®^ DNA/RNA Kit (Qiagen, Germantown, MD, USA) with slight modification involving a homogenizer versus agitation with a vortex to ensure separation of sample from the filter after concentration. For the enMF method, 600 μl of buffer PM1 and 6 μL of *β*-mercaptoethanol (Millipore Sigma, Burlington, MA, USA) were added into each bead beating tube. Bead beating tubes were homogenized using a Precellys^®^ Evolution 24 tissue homogenizer (Bertin Technologies, Rockville, MD, USA) under the following conditions: 3 × 20 s at 10,000 rpm at a 10 s interval. Following homogenization, tubes were centrifuged at 13,000 ×*g* for 1 min to pellet and extraction was completed according to manufacturer’s specifications. Samples concentrated by PEG precipitation and NPs were extracted according to manufacturer’s specifications. All extracted nucleic acid samples were eluted to a final volume of 100 μl and stored at −80 °C for downstream quantification and analysis.

#### Water quality testing

Additional grab samples were collected at the end of composite sample collection to perform physicochemical tests in the field including conductivity (μS/cm) via the Oakton CON 6+ Handheld Conductivity Meter (Cole-Parmer, Vernon Hills, IL, USA), temperature (°C), and pH via the Oakton pHTestr^®^ 50 Waterproof Pocket pH Tester 50 Series (Cole-Parmer, Vernon Hills, IL, USA), and free and total chlorine levels via the DR300 Pocket Colorimeter, Chlorine, Free + Total (Hach Company, Loveland, CO, USA). The reagents used for free and total chlorine were DPD Free Chlorine Reagent Powder Pillows, 10 ml (Hach Company, Loveland, CO, USA) and DPD Total Chlorine Reagent Powder Pillows, 10 ml (Hach Company, Loveland, CO, USA). Additional parameters including total organic carbon (TOC; mg/l), total organic nitrogen (TON; mg/l), total dissolved solids (TDS; mg/l), total suspended solids (TSS; mg/l), and biochemical oxygen demand (BOD; mg/l) were tested by an external environmental laboratory (Analytical Environmental Services, Inc., Atlanta, GA, USA).

#### Escherichia coli and total coliforms

*Escherichia coli* (*E. coli*) and total coliforms were detected and quantified using an IDEXX Colilert-18 assay kit (IDEXX Laboratories, Inc. Westbrook, ME, USA) according to manufacturer instructions, where *E. coli* fluoresced under UV light (6-W, 365 nm). A random composite (1 ml) of *E. coli*-positive Colilert-18 (IDEXX) samples (*n* = 24) was extracted from five positive wells, or 20% per plate, using a 3 ml syringe. Samples were stored at −80 °C in 25% glycerol for downstream analysis of AR genes.

#### AR screening

##### Culture-dependent:

Wastewater samples were screened for carbapenem-resistant Enterobacterales (CRE) and extended-spectrum *β*-lactamase (ESBL)-producing organisms using two selective chromogenic media, mSuperCARBA (mSC) (DRG International, Springfield, NJ, USA) for selection of CRE, and CHROMagar ESBL (DRG International, Springfield, NJ, USA) for selection of ESBL-producing Gram-negative bacteria. Phenotypic characterization of the isolates followed the mSC and ESBL selective agar product inserts, where *Klebsiella* spp., *Enterobacter*, spp. and *Citrobacter* spp. colonies were blue and *E. coli* colonies were pink. Frozen, *E. coli*-positive Colilert-18 samples (IDEXX) were thawed and 100 μl was either plated directly on mSC and CHROMagar ESBL (direct-plating) or inoculated in 5 ml tryptic soy broth (TSB) and incubated overnight at 35 °C (TSB-enriched) followed by plating 100 μl on mSC and CHROMagar ESBL. A random subset of all the colonies (i.e., mixture of typical and atypical colonies; *n* = 28) were selected for Matrix-Assisted Laser Desorption/Ionization Time-of-Flight (MALDI-TOF; MALDI Biotyper) (Bruker Daltonics, Billerica, MA USA) using the Bruker and CDC MicrobeNet databases (https://microbenet.cdc.gov/) to confirm identification (Suzuki et al. 2020).

##### Culture-independent:

Magnetic filter funnels (Pall Corporation, Port Washington, NY, USA) were mounted onto a multi-position filter manifold (MilliporeSigma, Burlington, MA, USA) and loaded with 0.45 μm pore-size, 47-mm diameter electronegative membrane filters (enMFs; Fisher Scientific, Waltham, MA, USA). Ten milliliters of wastewater was filtered (*n* = 23) and frozen (−80 °C) until DNA extraction using the DNeasy^®^ PowerWater^®^ kit (Qiagen, Germantown, MD, USA). The composite *E. coli*-positive Colilert-18 (IDEXX) samples (1 ml; *n* = 23) were extracted using the Thermal NaOH Bacterial Lysate method (CDC 2024). Extracted (filters) and lysed (positive *E. coli* Coliert-18 composites) samples were frozen (−20 °C) until multiplex real-time polymerase chain reaction (PCR) was performed. Carbapenemase-producing genes were identified using multiplex real-time PCR for the *bla*_KPC_, *bla*_NDM_, *bla*_VIM_, and *bla*_OXA-48_-like carbapenemase-producing genes by assays as previously described (CDC 2011; Lutgring et al. 2018; Prussing et al. 2021).

### Detection of SARS-CoV-2, process controls (BRSV, OC43), and endogenous controls (CrAssphage, PMMoV) by ddPCR

ddPCR assays for the detection and quantification of SARS-CoV-2 and its viral surrogates (BRSV, OC43, CrAssphage, and PMMoV) were performed using a Bio-Rad QX200 manual droplet generator system (Bio-Rad, Hercules, CA, USA). Nucleic acid extracts were used as a template and analyzed using One-Step RT-ddPCR Advanced Kit for Probes (Bio-Rad, Hercules, CA, USA) or ddPCR Supermix for Probes (No dUTP) kit (Bio-Rad, Hercules, CA, USA). SARS-CoV-2 and BRSV were detected using the 2019-nCoV CDC triplex probe assay (dEXS28563542, Bio-Rad, Hercules, CA, USA). OC43 and CrAssphage were detected using a singleplex assay. For the triplex assay, 8 μl of RNA was added to 5.5 μl of One-Step Advanced Supermix, 1.1 μl of 15 mM dithiothreitol (DTT), 2.2 μl of reverse transcriptase (RT), 1.1 μl of 20X triplex probe assay, and 4.1 μl RNase-free water in a final volume of 22 μl. For the singleplex assay used to detect OC43, 8 μl of RNA was added to 5.5 μl of One-Step Advanced Supermix, 1.1 μl of 300 mM DTT, 2.2 μl of RT, 1 μl of forward and reverse primers, 0.3 μl of probe (final concentrations were 900 nM of primers and 250 nM of probe), and 2.9 μl RNase-free water in a final volume of 22 μl. For the singleplex assay used to detect CrAssphage, 2 μl of DNA was added to 11 μl of ddPCR Supermix for Probes (No dUTP), 1 μl of forward and reverse primers, 0.3 μl of probe (final concentrations were 900 nM of primers and 250 nM of probe), and 6.7 μl nuclease-free water in a final volume of 22 μl. Sequences for all primers and probes used in this study are detailed in Table S1B (Supplementary Information) (Boxus et al. 2005; Dare et al. 2007; Stachler et al. 2017; Greaves et al. 2020; Lu et al. 2020). For droplet generation, 20 μl of reaction mixture and 70 μl of droplet generation oil were added to the droplet generator cartridges and droplets were formed using the droplet generator. Forty microliters of droplets were carefully transferred to a semi-skirted 96-well ddPCR plate, sealed using the PX1 Plate Sealer, and transferred to a C1000 touch thermal cycler. The amplification of the triplex assay and singleplex (OC43) were performed as follows: 1 cycle of 60 min at 50 °C and 10 min at 95 °C, 40 cycles of 30 s at 94 °C and 1 min at 55 °C, 1 cycle of 10 min at 98 °C and a 30 min hold at 4 °C. The amplification of the singleplex assay for CrAssphage was performed as follows: 1 cycle of 10 min at 95 °C, 40 cycles of 30 s at 94 °C and 1 min at 60 °C, 1 cycle of 10 min at 98 °C and a 30 min hold at 4 °C. All steps were run using a ramp rate of 2 °C/s.

Each run was performed using positive controls (synthetic 2019_nCoV RNA control (CDC), BRSV, OC43, CrAssphage, and PMMoV) and negative controls (extraction blanks and non-template controls). Technical triplicates were run for each sample including controls. To test for PCR inhibition, each template was run and analyzed using two dilution factors (undiluted and 2-fold) for all assays. After amplification, plates were transferred to the droplet reader. Reactions with less than 10,000 total droplets were repeated. Data were analyzed using QuantaSoft^™^ AnalysisPro Software and reported as genomic copies per microliter (gc/μl) of wastewater. For analysis, the mean of triplicate samples was determined. Samples were considered positive if >3 positive droplets were detected for SARS-CoV-2 N1 or N2 markers in 2 of 3 triplicate samples. Results were considered detected, but not quantifiable if there were <3 positive droplets in any of the triplicate samples, and negative if no droplets were detected. Final results were expressed as log_10_ genomic copies/microliter (log_10_ gc/μl) using Equation (1) (Graham et al. 2021; 2023).

### SARS-CoV-2 sequencing

One sample, positive for SARS-CoV-2 with a real-time PCR cycle threshold (*C*_t_) value of 29.7 was sequenced, where a *C*_t_ threshold of 31 has been determined for full genome sequence coverage. SARS-CoV-2 genome-tiling amplicons (300–500 bp long) were produced in a multiplex PCR reaction using template cDNA generated by reverse transcription with random hexamers as described previously (Paden et al. 2020). The genome sequences were obtained by next-generation sequencing using the Illumina DNA Library Prep kit strategy (Illumina Inc., San Diego, CA, USA) on a MiSeq instrument (Illumina Inc., San Diego, CA, USA). Additional sequencing was attempted using a separate set of longer amplicons (800–1,000 bp) with the Nanopore GridION Sequencer (Oxford Nanopore Technologies, Oxford, UK) as confirmation of template availability. Sequences were analyzed using the CFSAN Wastewater Analysis Pipeline (Kayikcioglu et al. 2023) with Freyja (version freyja, version 1.3.11) to identify variants; a list of typical mutations for SARS-CoV-2 variants was obtained from COV-Spectrum (Chen et al. 2022). Genome consensus sequences were generated through mapping reads to the SARS-CoV-2 Wuhan-Hu-1 genome (GenBank accession no. NC_045512.2) with the iterative refinement meta-assembler (Shepard et al. 2016). The sequences were analyzed by aligning each consensus genome with the Wuhan-Hu-1 genome sequence (Hall 1999). The available sections were compared with the known locations for determining a variant.

### Recovery efficiency and statistics

Recovery efficiency was calculated as described elsewhere (Graham et al. 2021; Graham et al. 2023), where any dilutions conducted for the RNA sample template prior to ddPCR are reflected in the ddPCR output ‘C’:

Equation #1

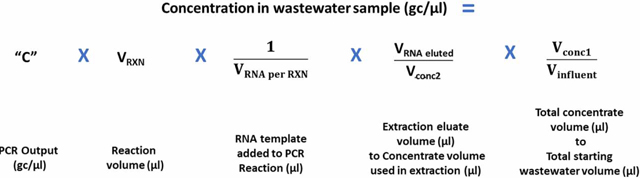



### Statistical analysis

Descriptive statistics were conducted on 10 water quality parameters to characterize water quality including pH, temperature (°C), conductivity (μS/cm), biological oxygen demand (BOD), total dissolved solids (TDS), total suspended solids (TSS), total organic nitrogen (TON), total organic carbon (TOC), total coliforms, and *E. coli* (Supplementary Information-Table S2). *E. coli* was not recovered from wastewater samples in two instances due to unknown facility factors, not related to measured water quality parameters and, as a result, were excluded from the analysis.

For the exogenous and endogenous controls, BRSV, PMMoV, and crAssphage, evaluated using three different methods, non-detects were substituted with half the minimum detected concentration prior to undergoing log_10_ transformation. The Shapiro–Wilk test was used to assess the normality of these datasets, indicating a non-normal distribution. Consequently, non-parametric methods were employed for further analysis. The Kruskal–Wallis test was used to compare recovered concentrations of BRSV, PMMoV, and crAssphage by three different concentration methods. A Dunn’s post-hoc test with Bonferroni adjustment was used to analyze pairwise differences between methods. All analyses and figures were produced using R software (version 4.2.2). A significance level (alpha) of 0.05 was set for all tests, and differences were considered significant when *p*-values were below this threshold.

## RESULTS

### Water quality, *E. coli*, and total coliforms

The wastewater parameters: pH, temperature, electrical conductivity, BOD, TDS, TSS, TOC, and TON are shown in Table S2 (*n* = 23) (Supplementary Information), where the means (standard deviation) were 10.1 (1.3), 24.1 °C (1.8), 432.5 μS/cm (205.7), 207.7 mg/l (203.1), 278.4 mg/l (155.4), 103.3 mg/l (87.6), 113.1 mg/l (71.2) and 14.7 mg/l (11.00), respectively. Culture-dependent analysis using Colilert-18 demonstrated the presence of *E. coli* (5.0 log_10_ MPN/100 ml; 95% CI 2.1 to 6.4 log_10_ MPN/100 ml) and total coliforms (6.0 log_10_ MPN/100 ml; 95% CI 2.9 to 7.4 log_10_ MPN/100 ml). *E. coli* was not significantly correlated with any water quality parameters (*n* = 21; r_s_ < 0.42, *p*-value > 0.05). Total coliforms and *E. coli* concentrations over time for each sampling date are illustrated in Supplementary Figure S1.

### AR screening

**Figure 1 |:**
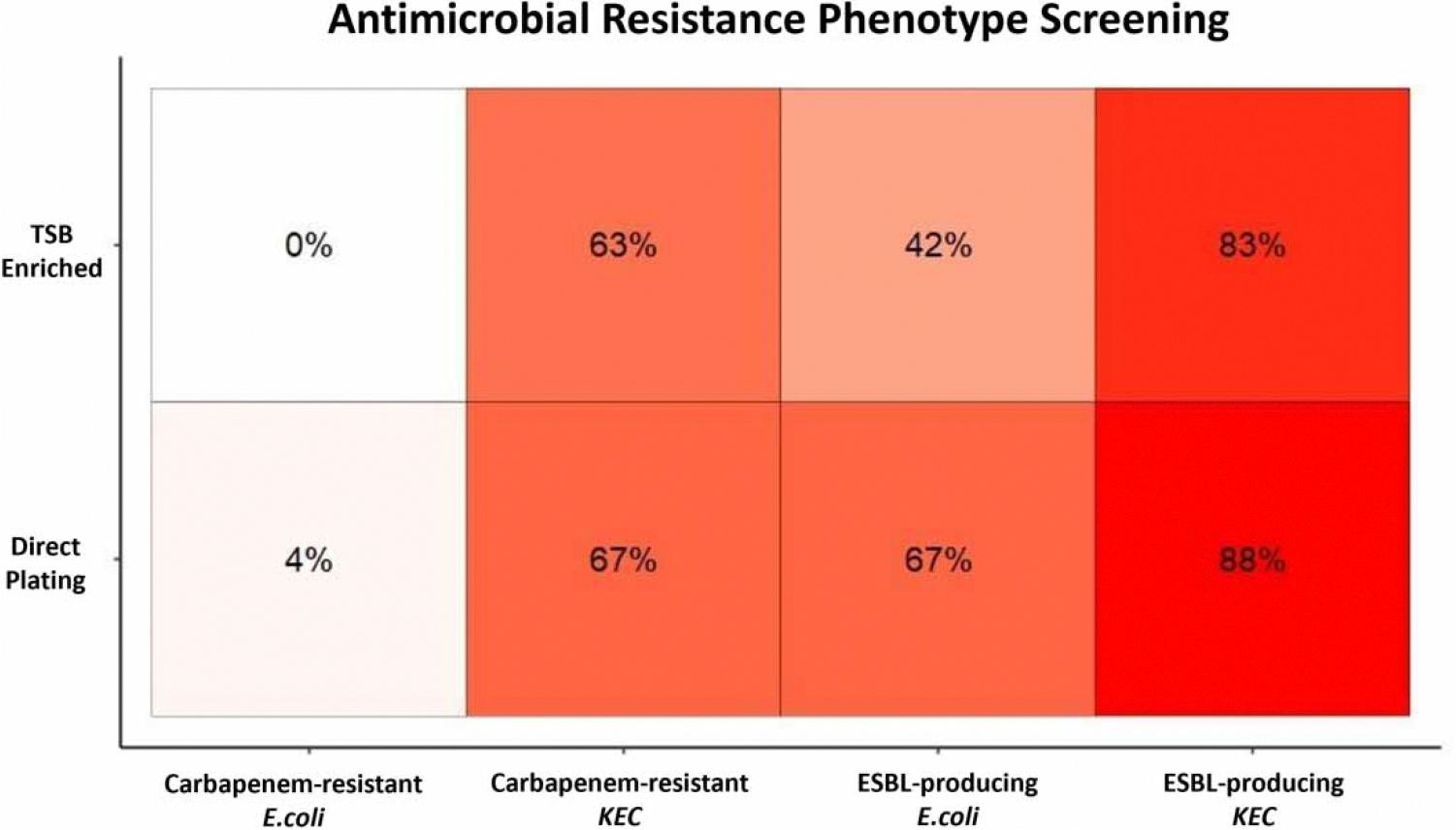
Phenotypic screening for carbapenem-resistant (CRE) and ESBL-producing Enterobacterales from wastewater samples collected at a single SNF between September 2021 to November 2021. Values are presented as percent of total Colilert-18 *E. coli* positive isolates (*n* = 24) recovered from selective agar media. Isolates were *E. coli* and KEC genera and were either plated directly on selective media from IDEXX frozen stocks or enriched in tryptic soy broth (TSB) prior to plating on selective media.

The percentage of *E. coli*-positive Colilert-18 (IDEXX) composites exhibiting an antimicrobial-resistant phenotype on the selective agars are summarized in Figure 1. The highest percentages were observed in the ESBL-producing *Klebsiella*, *Enterobacter*, and *Citrobacter* (KEC) species at 88% (21 of 24) and 83% (20 of 24) for direct plating and TSB-enriched methods, respectively. ESBL-producing *E. coli* isolates were observed in 67% (direct plating; 16 of 24) and 42% (TSB-enriched; 10 of 24) of samples. Carbapenem-resistant KEC were observed in 67% (direct plating; 16 of 24) and 63% (TSB-enriched; 15 of 24) of samples. Finally, the least observed isolates were the carbapenem-resistant *E. coli* at 4% (direct plating; 1 of 24) and 0% (TSB-enriched; 0 of 24). Overall, MADLI-TOF confirmed the following species were identified on mSC agar: *Aeromonas hydrophilia* (*n* = 1), *E. coli* (*n* = 1), *Klebsiella pneumoniae* (*n* = 1); whereas the following were identified on CHROMagar ESBL: *E. coli* (*n* = 4), *Klebsiella pneumoniae* (*n* = 1), *Enterobacter bugandensis* (*n* = 3), *Enterobacter asburiae* (*n* = 1), *Raoultella ornithinolytica* (*n* = 1), *Citrobacter braakii* (*n* = 1), *Citrobacter freundii* (*n* = 1), *Providencia stuartii* (*n* = 1), and *Klebsiella oxytoca* (*n* = 1).

Eighteen (78%) of 23 filtered wastewater samples were positive for the *bla*_KPC_ gene, while all samples were negative for *bla*_NDM_, *bla*_VIM_, and *bla*_OXA-48_-like genes (Lenz et al. 2025). No carbapenemase-producing genes were detected in the 23 *E. coli*-positive Colilert-18 (IDEXX) composites.

### Detection and quantification of BRSV and OC43 by RT-ddPCR

**Figure 2 |:**
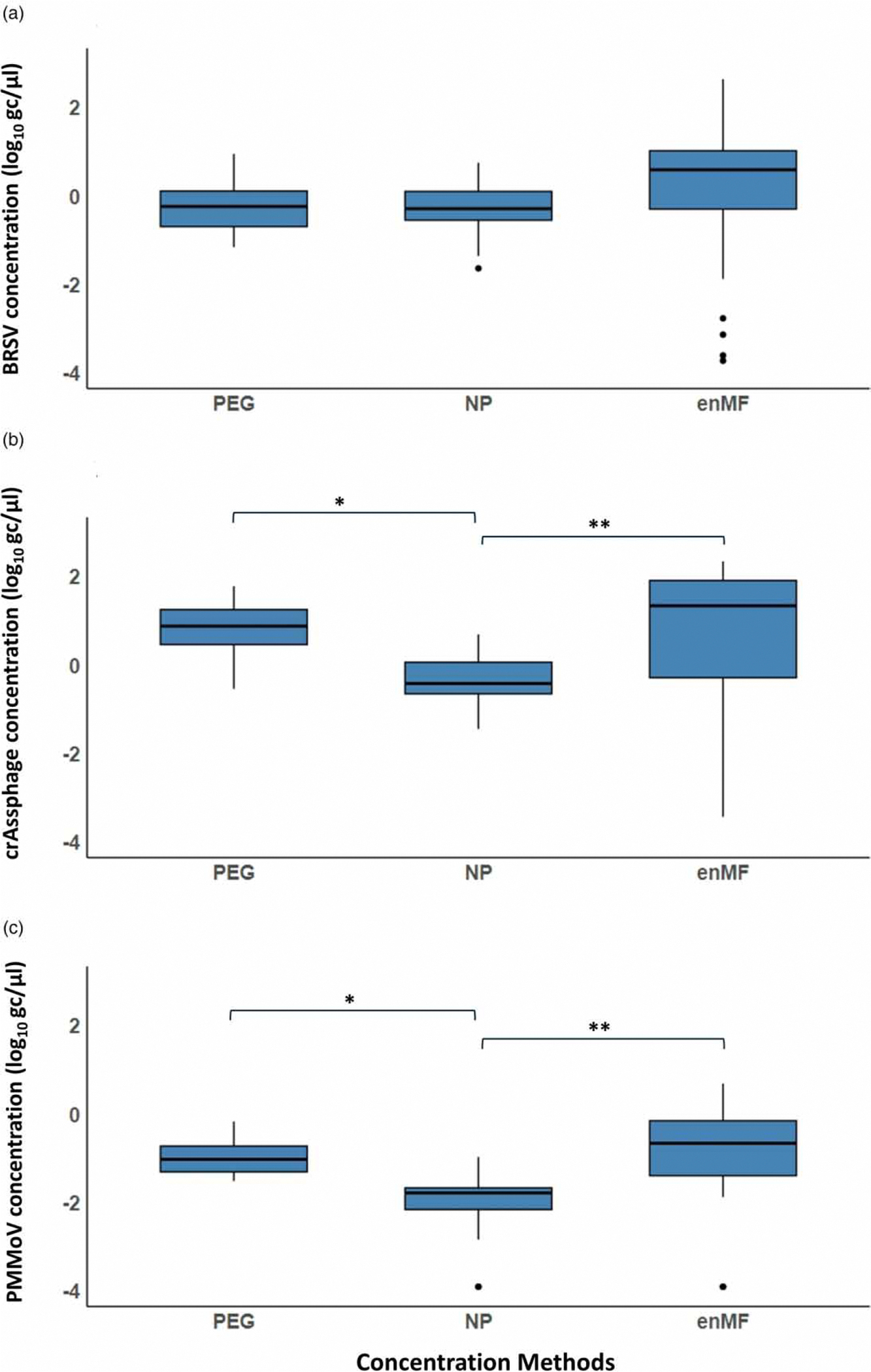
(a)–(c) Recovery of exogenous and endogenous controls spiked into SNF wastewater samples using polyethylene glycol (PEG) precipitation, nanoparticles (NP), and electro-negative membrane filtration (enMF) concentration methods for (a) BRSV (log_10_ genome copies per μl), (b) cross-assembly phage (crAssphage; log_10_ genome copies per μl), and (c) pepper mild mottle virus (PMMoV; log_10_ genome copies per μl). Values are log_10_ normalized medians ± standard deviation (*n* = 23). In these box plots, center lines represent medians, box limits indicate the 25th and 75th percentiles, and whiskers extend 1.5 times the interquartile range. Data points beyond this range are outliers. Asterisks indicate significant differences between concentration methods ( *p*-value < 0.005). Wastewater samples were collected from a single SNF from September 2021–November 2021.

The recovered concentrations of BRSV by method are shown in Figure 2(a). The highest concentrations recovered were observed for enMF, with a median concentration of 0.60 log_10_ gc/μl wastewater (*n* = 23). NP and PEG yielded median concentrations of −0.28 log_10_ gc/μl and −0.23 log_10_ gc/μl (*n* = 23), respectively. The average volume contained within each droplet was 0.85 nL (Pinheiro et al. 2012). The fraction of positive droplets ranged from 0 to 0.9905. A Shapiro–Wilk test indicated that our data did not meet the assumption of normality. A non-parametric analysis using a Kruskal–Wallis test indicated no significant difference between the concentration method groups (H(2) = 4.97, *p*-value = 0.08). The median (min-max) percent recoveries for BRSV were as follows: 69.84% (0.004–948.75%) for enMF, 13.02% (1.27–70.86%) for NP, and 12.88% (1.64–112.00%) for PEG.

Median (min-max) percent recoveries of the extraction control, OC43, by the method were 1.31% (0.00–3.68%), 1.50% (0.66–2.73%), and 1.53% (0.75–3.92%) for enMF, NP, and PEG (*n* = 23), respectively.

### Endogenous virus controls and concentration methods

CrAssphage was detected at median concentrations (min-max) of 0.89 (−0.53 to 1.80) log_10_ gc/μl, −0.41 (−1.44 to 0.70) log_10_ gc/μl, and 1.35 (−3.60 to 2.35) log_10_ gc/μl for PEG, NP, and enMF, respectively (*n* = 23) (Figure 2(b)). A non-parametric analysis using a Kruskal–Wallis test indicated a significant difference in crAssphage detection between the concentration method groups (H(2) = 20.41, *p*-value < 0.00005). A Dunn’s post-hoc test with Bonferroni adjustment was used to further analyze the pairwise differences between methods. Significant differences were observed between enMF and NP (*Z* = 4.06, *p*-value < 0.005) and between NP and PEG (*Z* = −3.74, *p*-value < 0.005). However, the comparison between enMF and PEG was not statistically significant (*Z* = 0.323, *p*-value = 1.0).

PMMoV was detected at median concentrations (min-max) of −1.02 (−1.51 to −0.16) log_10_ gc/μl, −1.75 (−2.87 to −0.97) log_10_ gc/μl, and −0.42 (−1.88 to 0.70) log_10_ gc/μl for PEG, NP, and enMF respectively (*n* = 23) (Figure 2(c)). A non-parametric analysis using a Kruskal–Wallis test indicated a significant difference in PMMoV detection between the concentration method groups (*H*(2) = 26.62, *p*-value < 0.000005). A Dunn’s post-hoc test with Bonferroni adjustment was used to further analyze the pairwise differences between methods. Significant differences were observed between enMF and NP (*Z* = 4.72, *p*-value < 0.005) and between NP and PEG (*Z* = −4.17, *p*-value < 0.005). However, the comparison between enMF and PEG was not statistically significant (*Z* = 0.55, *p*-value = 0.87).

### SARS-CoV-2 detection

SARS-CoV-2 was detected and quantified from an enMF-concentrated wastewater sample on 26 October 2021 (week 6) at −1.36 log_10_ gc/μl and −1.56 log_10_ gc/μl for the N1 and N2 gene primers, respectively. Questionnaire data from the corresponding week indicated a single known case, isolated within the COVID-19 unit, who was able to use the toilet during the week of wastewater surveillance and NHSN data confirmed cases in two residents and one staff. CrAssphage was detected at 1.68 log_10_ gc/μl. Sequencing confirmed the presence of SARS-CoV-2, but the specific variant cannot be confidently established. Illumina sequencing of pooled 300–500 bp amplicons and Nanopore sequencing of separate 800–1,000 bp amplicons both recovered approximately 35% of the SARS-CoV-2 genome; the same regions of the genome were obtained from each sequencing attempt. The available sequence contained only a single complete gene, ORF3a. The Delta variant is assigned based on variation at 29 sites across 8 genes (Chen et al. 2022). With only a single complete gene present, it was not possible to confidently identify the variant in the sample. This sample likely does not contain enough SARS-CoV-2 genomic material to obtain a complete genome sequence, as the second, long-amplicon sequencing attempt did not provide any further information. Additional week-by-week questionnaire epidemiological data related to SARS-CoV-2 cases in the facility, toilet use, and wastewater SARS-CoV-2 detection is provided as Supplementary Information (Table S3) (CDC 2021).

## DISCUSSION

The importance of WBE as a public health monitoring tool was highlighted during the height of the COVID-19 pandemic (Gamage et al. 2024). SNFs and other long-term care facilities were heavily impacted by the pandemic, highlighting the potential role that wastewater surveillance, coupled with clinical testing, could play in detecting transmission and informing infection control measures. The wastewater surveillance study by Gamage et al. (2024) highlighted the utility of WBE over eight distinct facilities and demonstrated how in some cases wastewater surveillance served as an early indicator of asymptomatic or presymptomatic SARS-CoV-2 residents; however, they noted that it was not reliable enough to replace other established practices (Gamage et al. 2024). Keck et al. (2024) suggest that ‘such testing (wastewater) could provide an early warning to trigger enhanced clinical testing or infection prevention activities’ (Keck et al. 2024). To that end, protocols should be developed carefully as factors such as sample type, frequency of collection, concentration methods, and type of analytical detection (e.g., RT-PCR vs ddPCR) can impact the reproducibility of wastewater surveillance results (Pecson et al. 2021). Establishing clear standard operating procedures that incorporate quality control parameters along with optimal method sensitivity is also important (Pecson et al. 2021).

Additionally, the characteristics of SNF wastewater effluents and their associated water quality parameters (e.g., pH, temperature, BOD, and TSS) should be considered, as they may have an impact on the detection of microbial targets. In this study, these parameters did not appear to greatly impact the recovery of endogenous controls in the wastewater likely due to the short residence time (i.e., minutes) from the point of flush to the point of sampling. Taking the factors of variable front-end concentration methods, macro-pollutants, and small populations into consideration, this study successfully demonstrated the detection of SARS-CoV-2, endogenous controls, and antimicrobial-resistant genes/organisms at the SNF level.

A review by Achak et al. (2021) highlighted how hospital wastewater can be a source of both micro- and macro-pollutants, including pathogenic microorganisms which can be a source of disease transmission (Achak et al. 2021). The physicochemical properties of facility wastewater effluents could provide insight into the disease burden at a given facility; therefore, there is value in measuring water quality parameters. In this study, for example, measured values for conductivity, BOD, TSS, total coliforms, and *E. coli* fell within the ranges previously reported for hospital and urban wastewaters (Verlicchi et al. 2012). It should be noted that any cleaning or disinfection interventions facility personnel undertake could impact the recovery of target microorganisms. It was also noted that recovery of *E. coli* was impacted in at least two of our samples, and despite discussions with facility administrators and personnel, no external facility factors could be attributed to these occurrences including the water quality parameters measured. Future use of facility-level data can be helpful in understanding how facility signals align with community-level contributions.

One of the primary aims of this study was to determine which wastewater concentration method would provide the best recovery with minimal processing time. The concentration methods evaluated included traditional methods (e.g., enMF and PEG precipitation) and a more novel approach utilizing magnetic NPs. The recovery of both endogenous and exogenous viral targets using all three methods, combined with ddPCR for detection, suggested that each of the methods was a viable option for the wastewater surveillance approach in this study. When it came to detecting the exogenous control BRSV, we noted no statistical difference between enMF, PEG, and NP. However, the percent recovery range for enMF was greater than those observed for PEG and NP. Unlike crAssphage and PMMoV, BRSV is spiked into the wastewater samples and has a relatively short residence time in the wastewater matrix before concentration. It is possible that this difference in residence time, compared to the endogenous controls, may have resulted in no clear distinction between the three methods tested. Notably, the single positive SARS-CoV-2 sample in this study was recovered by enMF, but not from the other two concentration methods, suggesting that enMF may provide a bit more flexibility in its ability to capture a target organism, particularly when its presence in the wastewater is highly variable or limited. Unfortunately, with only a single positive SARS-CoV-2 sample and several samples that were detectable but not quantifiable (DNQ), the data was insufficient to conduct a proper analysis of the sensitivity and specificity of these methods. The results of this study do not necessarily point to enMF performing the best; however, taking into account that it was the only method that yielded a sample with detectable and quantifiable genetic material from SARS-CoV-2, and it provided a relatively quick and efficient workflow, made it stand out among the methods tested. Notably, rapid turnaround times and streamlined workflows were also observed for the NP method. Thus, for future wastewater surveillance studies, these concentration methods may be considered.

Wastewater surveillance of AR bacteria and genes was of particular interest to this study to understand their presence in the SNF population, particularly during clinical presentations of SARS-CoV-2 early in the pandemic. One of the goals was to pilot selective agars, typically used for clinical screening, to screen for multi-drug-resistant organisms in wastewater, as well as evaluate front-end approaches from filtered or enriched samples directly to PCR from SNF wastewater. Various clinically and environmentally relevant bacterial isolates, exhibiting carbapenem-resistant and extended-spectrum *β*-lactamase-producing phenotypes, were successfully isolated and confirmed using MALDI-TOF. The TSB enrichment after Colilert-18 was not beneficial in increasing carbapenem-resistant or ESBL-producing phenotype detection as compared to direct plating.

The molecular detection of the *bla*_KPC_ gene from filtered wastewater samples via PCR was successful, whereas *E. coli*-positive Colilert-18 samples did not yield any carbapenem resistance gene detection. It should be noted that the filters represented 10 ml of wastewater, went through a more robust extraction process, and did not bias growth. Although molecular detection of resistance genes was achieved from filtered wastewater samples, a limitation of this study is the lack of resistance mechanism confirmatory PCR for isolates exhibiting AR phenotypes. Future studies should include this confirmatory PCR testing to determine if the resistance mechanism is present in the isolates and also correlate it to available facility-level clinical data. This study relied on both culture- and non-culture-based methods that were compatible with the facility sampling schedule, sample storage capacities, and laboratory workflow. An examination of the Antimicrobial Resistance and Patient Safety Portal data of the percent AR from healthcare-associated infection types (i.e., catheter-associated urinary tract infections, central line-associated bloodstream infections, surgical site infections) in Georgia in 2021 indicated that 7.8 and 6.6% were associated with carbapenem-resistant *Klebsiella* and *Enterobacter*, respectively, whereas only 0.8% were associated with carbapenem-resistant *E. coli* (CDC 2023b). Although recovered from an environmental source and not directly from patients, in this study a greater percentage of the isolates that exhibited a carbapenem-resistant phenotype were in the *Klebsiella* and *Enterobacter* genera and a much lower percentage was observed for *E. coli*. While the AR data presented here and the application of wastewater surveillance at healthcare facilities is nascent, evaluating existing specimen approaches for wastewater samples is a feasible starting place. Expanding these approaches to complement existing patient care data and infection control practices could bridge knowledge gaps and challenges raised by public health agencies (CDC 2022).

Similarly, this study also provided some insight into the relationship between real-time, patient case data and the ability of wastewater surveillance to capture corroborating data on the levels of viral circulation in specific populations. Although linking sequencing data from wastewater samples to positive clinical samples was beyond the scope of this study and limited by the facility’s capacity at the time, it is interesting to note that during the same period (September–November 2021), data provided by reporting facilities to both Centers for Medicare & Medicaid Services and the Georgia Department of Public Health (GADPH) indicated reductions in both COVID-19 cases and hospitalizations among nursing home residents (CMMS 2021). For example, according to the GADPH, rolling 7- and 14-day percent positive thresholds decreased by nearly 50% from month to month (September–November 2021), indicating a reduction in SARS-CoV-2 infections during this period. During our study period, only one of eighteen composite wastewater samples had detectable levels of SARS-CoV-2 that could be quantified by ddPCR. Sequencing data confirmed the presence of SARS-CoV-2 but could not confidently assign the variant, although the circulating variant at the time in Georgia was delta. In several instances there were samples that were DNQ, suggesting that additional factors such as waste discharge from the facility and variable shedding rates, including from healthcare workers and visitors, could impact the levels of virus that can be detected and quantified. Furthermore, at the time, the SARS-CoV-2 ddPCR kit used for detection had been granted Emergency Use Authorization for the qualitative detection of nucleic acids from SARS-CoV-2, from nasal swab specimens, and may have been expanded beyond its scope. Based on our findings and the reporting of other groups, wastewater-based surveillance would be sensitive enough to detect both increases and decreases associated with pathogen transmission among the SNF population (Fernandez-Cassi et al. 2021; D’Aoust et al. 2021).

## CONCLUSION

Overall, this study successfully demonstrated the detection of SARS-CoV-2, endogenous controls, and antimicrobial-resistance genes/organisms at the SNF level. Future efforts using the confirmed methodological workflow from this study will be expanded to include additional SNFs to establish best practices and longitudinal measurements needed to correlate SARS-CoV-2 in wastewater with COVID-19 resident infections. Wastewater surveillance provides unique insight into the spread of existing and emerging disease-causing pathogens in a given population and can help to inform infection control strategies in real-time and long-term public health policies. While the COVID-19 pandemic presented unique challenges to the existing public health framework, it also provided an opportunity to evaluate the potential strengths and weaknesses of wastewater surveillance applications at SNFs.

## Supplementary Material

Supplementary Material

## Data Availability

All relevant data are included in the paper or its Supplementary Information.

## HIGHLIGHTS

- Wastewater surveillance methods were successfully applied at a skilled nursing facility.
- Electronegative membrane filtration resulted in the highest viral recovery.
- Carbapenem resistance genes were detected in wastewater samples using clinical assays.
