# Supplementary material for: Skilled nursing facility wastewater surveillance: a SARS-CoV-2 and antimicrobial resistance detection pilot study
Source: J Water Health. Author manuscript; Available in PMC 2025 Aug 5. (PMC12323735; doi:10.2166/wh.2025.374)
Supplement: Supplementary Material [file NIHMS2096720-supplement-Supplementary_Material.docx]

**Supplementary Information**

**Table S1A**: Endogenous controls used in this study

| **Controls** | **Sequences of control (5’-3')** |
| --- | --- |
| **CrAssphage CPQ56 (146 bp)** | AGGCTAAGCTCAGAAGTACAAACTCCTAAAAAACGTAGAGGTAGAGGTATTAATAACGATTTACGTGATGTAACTCGTAAAAAGTTTGATGAACATACTGATTGTAATAAAGCTAATGGCTTGTTTATTGGTCATCCTCGATAGCT |
| **PMMoV (78 bp)** | AAUGAGAGUGGUUUGACCUUAACGUUUGAGCGGCCUACCGAAGCAAAUGUCGCACUUGCAUUGCAACCGACAAUUGCA |

**Table S1B**: Primers/probes used for the detection of severe acute respiratory syndrome coronavirus 2 (SARS-CoV-2), bovine respiratory syncytial virus (BRSV), pepper mild mottle virus (PMMoV), human coronavirus OC43 (HCoV OC43), and the cross-assembly phage *Carjivirus communis* (CrAssphage). Wastewater samples were collected from a single skilled nursing facility (SNF) from September 2021-November 2021.

| **Target** | **Primer/Probes** | **Sequence** | **Amplicon Size** **(bp)** | | **Annealing Temp (°C)** | **Working Conc.** | **Ref.** |
| --- | --- | --- | --- | --- | --- | --- | --- |
| SARS-CoV-2 (CDC N1) | Fwd | 5’-GACCCCAAAATCAGCGAAAT-3’ |  |  | | 900 | (Lu *et al.*, 2020) |
|  | Rvs | 5’-TCTGGTTACTGCCAGTTGAATCTG-3’ | 72 | 55 | | 900 |  |
|  | Probe | <FAM>ACCCCGCATTACGTTTGGTGGACC<3IABkFQ> |  |  | | 250 |  |
| SARS-CoV-2 (CDC N2) | Fwd | 5’-TTACAAACATTGGCCGCAAA-3’ |  |  | | 900 | (Lu *et al.*, 2020) |
|  | Rvs | 5’-GCGCGACATTCCGAAGAA-3’ |  |  | | 900 |  |
|  | Probe 1 | <FAM>ACAATTTGCCCCCAGCGCTTCAG<3IABkFQ> | 67 | 55 | | 250 |  |
|  | Probe 2 | <HEX>ACAATTTGCCCCCAGCGCTTCAG<3IABkFQ> |  |  | | 250 |  |
| Bovine respiratory syncytial virus (BRSV) | Fwd | 5’-GCAATGCTGCAGGACTAGGTATAAT-3’ |  |  | | 900 | (Boxus *et al.*, 2005) |
|  | Rvs | 5’-ACACTGTAATTGATGACCCCATTCT-3’ | 124 | 55 | | 900 |  |
|  | Probe | <5HEX/ACCAAGACTTGTATGATGCTGCCAAAGCA<3IABkFQ> |  |  | | 250 |  |
| Human coronavirus (HCoV OC43) | Fwd | 5’-CGATGAGGCTATTCCGACTAGGT-3’ |  |  | | 900 | (Dare *et al.*, 2007) |
|  | Rvs | 5’-CCTTCCTGAGCCTTCAATATAGTAACC-3’ | 76 | 55 | | 900 |  |
|  | Probe | <5FAM/TCCGCCTGGCACGGTACTCCCT<3IABkFQ> |  |  | | 250 |  |
| Pepper mild mottle virus (PMMoV) | Fwd | 5’-GAGTGGTTTGACCTTAACGTTGA-3’ |  |  | | 900 | (Greaves *et al.*, 2020) |
|  | Rvs | 5’-TTGTCGGTTGCAATGCAAGT-3’ | 68 | 60 | | 900 |  |
|  | Probe | <5HEX/AGGCCTACCGAAGCAAATGTCG<3IABkFQ> |  |  | | 250 |  |
| CrAssphage (CPQ56) | Fwd | 5’-CAGAAGTACAAACTCCTAAAAAACGTAGAG-3’ |  |  | | 900 | (Stachler *et al.*, 2017) |
|  | Rvs | 5’-GATGACCAATAAACAAGCCATTAGC-3’ | 125 | 60 | | 900 |  |
|  | Probe | <5FAM/AATAACGATTTACGTGATGTAAC<3IABkFQ> |  |  | | 250 |  |

**Table S2:** Descriptive statistics of water quality indicators from wastewater samples collected from a single skilled nursing facility (SNF) from September 2021-November 2021.

| **Parameter** | **n** | **Mean** | **Std Dev** | **Min** | **Median** | **Max** |
| --- | --- | --- | --- | --- | --- | --- |
| **pH** | 23 | 10.1 | 1.3 | 7.21 | 10.6 | 11.9 |
| **Temperature (°C)** | 23 | 24.1 | 1.8 | 21.2 | 23.9 | 29.7 |
| **Electrical Conductivity (µS/cm)** | 23 | 432.5 | 205.7 | 179.5 | 408.1 | 910.7 |
| **BOD (mg/l)** | 23 | 207.7 | 203.1 | 50.4 | 156 | 1,040.00 |
| **TDS (mg/l)** | 23 | 278.4 | 155.4 | 78 | 257 | 765 |
| **TSS (mg/l)** | 23 | 103.3 | 87.6 | 25 | 83.8 | 406 |
| **TOC (mg/l)** | 23 | 113.1 | 71.2 | 35.9 | 83.4 | 250 |
| **TON (mg/l)** | 23 | 14.7 | 11 | 5.1 | 11.6 | 52.3 |
| **Total Coliform (Log_10_ MPN/100 ml)** | 23 | 6 | 1.2 | 2.9 | 6.2 | 7.4 |
| ***E. coli* (Log_10_ MPN/100 ml)** | 21* | 5 | 0.9 | 2.1 | 4.9 | 6.4 |

The wastewater parameters pH, temperature (°C), electrical conductivity (µS/cm), biochemical oxygen demand (BOD), total dissolved solids (TDS), total suspended solids (TSS), total organic carbon (TOC), total organic nitrogen (TON), total coliform (log_10_ MPN/100 ml) and *Escherichia coli* (*E. coli*; log_10_ MPN/100 ml) were measured. *Sample size (n) is 23 with the exception of *E. coli*, which had a sample size of 21. Values are mean, standard deviation (std dev), minimum (min), median, and maximum (max). Wastewater samples were collected from a single skilled nursing facility (SNF) from September 2021-November 2021.

**Figure S1:** Detection of total coliform (TC) and *Escherichia coli* (*E. coli*) from wastewater samples collected at a single skilled nursing facility (SNF) between September 2021 to November 2021 (n=23). Values are presented as log_10_ MPN/ 100 ml of TC (navy blue bar) and *E. coli* (light blue bar); the red diamond indicates dates where *E. coli* was not detected.


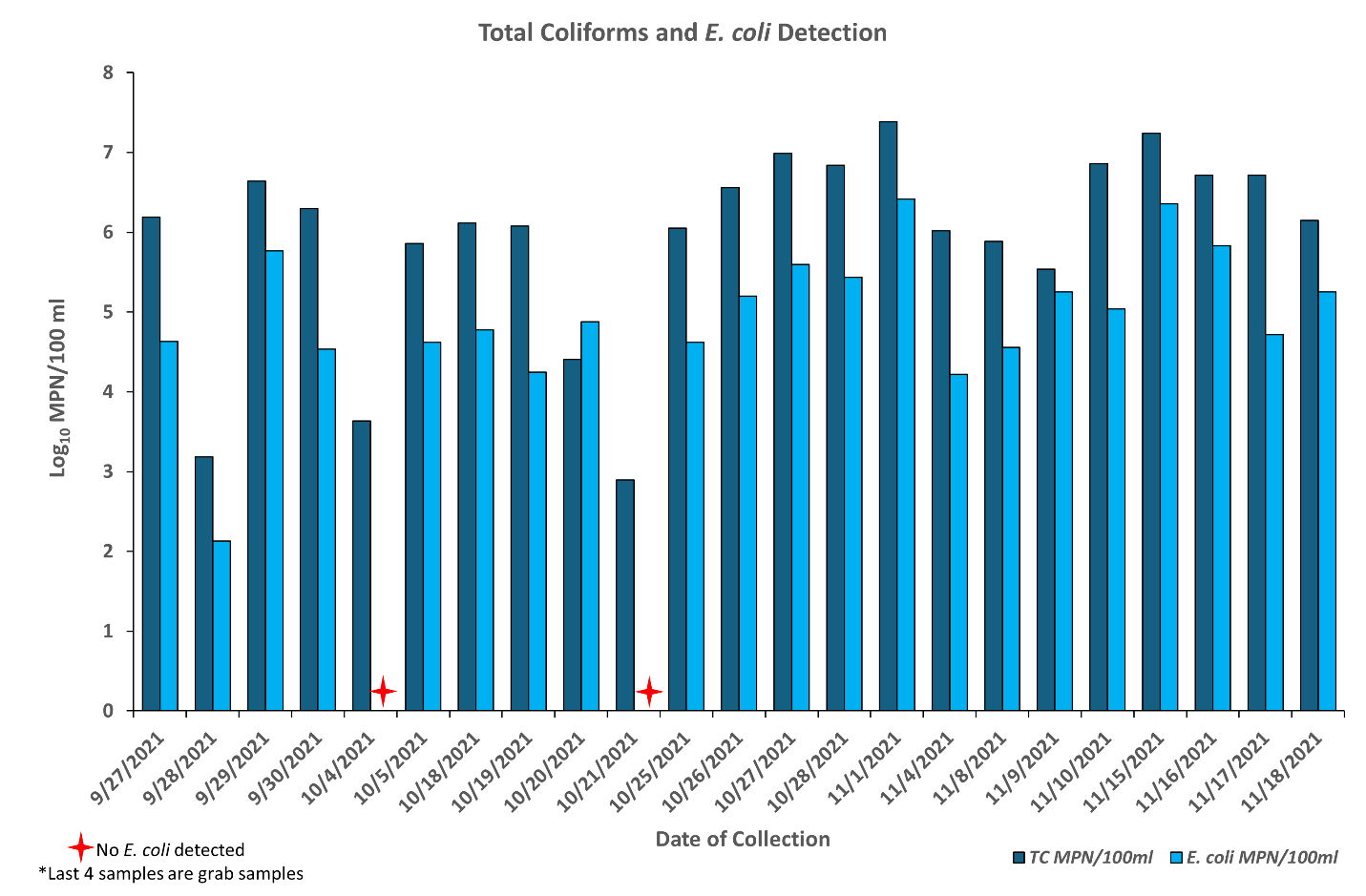


Supplementary Epidemiological Information

During the eight-week period of this study, SARS-CoV-2 was present at detectable levels during five of the eight weeks where wastewater samples were collected (Table S3). During the same period, five of the eight weekly questionnaires were completed and reported that for each week, at least one COVID-19 infected resident (active or within 30 days of confirmed SARS-CoV-2 infection) was able to use the toilet, thereby contributing human excrement to the wastewater. In the three weeks without documented toilet use (i.e., no completed weekly questionnaire for weeks 4, 8, 9), the following occurred: (1) one week experienced equipment issues resulting in no wastewater sample taken, thus no information regarding toilet use and data regarding SARS-CoV-2 detection was collected, and (2) one of the other two weeks resulted in wastewater samples with detectable but not quantifiable SARS-CoV-2. In four of the five weeks with completed questionnaires, SARS-CoV-2 was consistently detected (but not quantifiable) by the enMF and ddPCR workflow when at least one known COVID-19 infected resident was using the toilet within the same week. One week where the questionnaire was not completed (week 4), SARS-CoV-2 was not detected in wastewater while confirmed cases were reported in NHSN.

**Table S3:** Summary of SARS-CoV-2 detection, confirmed COVID-19 cases and resident toilet use during a wastewater surveillance pilot study conducted at a single skilled nursing facility (SNF) from September 2021-November 2021.

|  |  |  | **Residents using the toilet in relation to COVID-19 infection ^3^** | | | **Total** |
| --- | --- | --- | --- | --- | --- | --- |
|  | **SARS-CoV-2 Detection ^1^** | **Confirmed Cases ^2#^** | **Active Infection ^4^** | **Within 30-day Recovery ^5^** | **No Known Infection** | **Toileting** |
| **Week 1** | N/A | Yes (1 staff) | *Not captured with enrollment survey* | | | 66 ^7^ |
| **Week 2** | Yes (DNQ) | None | 1 | 9 | 31 | 41 |
| **Week 3** | No | None | 1 | 4 | 25 | 30 |
| **Week 4** | No | Yes (4 residents) | *Questionnaire was not completed, and no wastewater sample taken (equipment issue)* | | | |
| **Week 5** | Yes (DNQ) | Yes (6 residents) | 1 | ND ^6^ | ND | ND |
| **Week 6** | Yes (~-1.5 log_10_ gc/µl) | Yes (2 residents, 1 staff) | 1 | 0 | ND | ND |
| **Week 7** | Yes (DNQ) | Yes (4 residents) | 1 | ND | ND | ND |
| **Week 8** | No | None | *Questionnaire was not completed, and follow-up calls were not successful (staff transition)* | | | |
| **Week 9** | Yes (DNQ) | Yes (1 staff) | *Questionnaire was not completed, and follow-up calls were not successful (staff transition)* | | | |

^1^ SARS-CoV-2 detection in SNF wastewater: non-detects, detected but not quantifiable (DNQ), quantifiable (log_10_ gc/µl) by any assay workflow and at least one gene (N1, N2).

^2^ Confirmed Cases: Staff or resident, reported in NHSN and could represent delayed reporting as compared to completed questionnaires (CDC, 2021). ^#^ For staff, once tested positive - stayed out of work for 10 days.

^3^ Residents using toilet: Captured through the weekly questionnaire completed by facility administrators.

^4^ Active infection: Those residents within 10 days of infection, quarantined in a level 1 unit, and able to use the toilet.

^5^ Within 30 day recovery: Those residents now living in the general population after recovery from COVID-19.

^6^ ND means no data because this portion of the questionnaire was not completed.

^7^ Week 1, enrollment survey of 66 using the toilet in the facility is based upon the estimated 60% of 164 residents being diapered (98/164) [164 residents – 98 diapered = 66 toileted].

**Reference**

Lu, X., Wang, L., Sakthivel, S. K., Whitaker, B., Murray, J., Kamili, S., Lynch, B., Malapati, L., Burke, S. A., Harcourt, J., Tamin, A., Thornburg, N. J., Villanueva, J. M. & Lindstrom, S. 2020. US CDC Real-Time Reverse Transcription PCR Panel for Detection of Severe Acute Respiratory Syndrome Coronavirus 2. *Emerg Infect Dis,* 26**,** 1654-1665.

Boxus, M., Letellier, C. & Kerkhofs, P. 2005. Real Time RT-PCR for the detection and quantitation of bovine respiratory syncytial virus. *J Virol Methods,* 125**,** 125-130.

Dare, R. K., Fry, A. M., Chittaganpitch, M., Sawanpanyalert, P., Olsen, S. J. & Erdman, D. D. 2007. Human coronavirus infections in rural Thailand: a comprehensive study using real-time reverse-transcription polymerase chain reaction assays. *J Infect Dis,* 196**,** 1321-1328.

Greaves, J., Stone, D., Wu, Z. & Bibby, K. 2020. Persistence of emerging viral fecal indicators in large-scale freshwater mesocosms. *Water Research X,* 9**,** 100067.

Stachler, E., Kelty, C., Sivaganesan, M., Li, X., Bibby, K. & Shanks, O. C. 2017. Quantitative CrAssphage PCR Assays for Human Fecal Pollution Measurement. *Environ Sci Technol,* 51**,** 9146-9154.

CDC. 2021. *National Healthcare Safety Network (NHSN) long-term care facility component—HCP & resident COVID-19 vaccination.* [Online]. https://www.cdc.gov/nhsn/ltc/weekly-covid-vac/index.html. [Accessed 11/21/2021 2021].
